# Examining contextual influences on the service needs of homeless and unstably housed domestic violence survivors

**DOI:** 10.1002/jcop.22637

**Published:** 2021-06-19

**Authors:** Danielle Chiaramonte, Kathryn A.V. Clements, Gabriela López‐Zerón, Oyesola Oluwafunmilayo Ayeni, Adam M. Farero, Wenjuan Ma, Cris M. Sullivan

**Affiliations:** ^1^ Department of Psychiatry Yale University School of Medicine New Haven Connecticut USA; ^2^ Psychology Department Michigan State University East Lansing Michigan USA; ^3^ Center for Statistical Training and Consultation Michigan State University East Lansing Michigan USA

**Keywords:** help seeking, housing, intimate partner violence, latent class analysis

## Abstract

Domestic violence (DV) is a leading cause of homelessness for women, yet many DV agencies are just beginning to focus on helping clients stabilize their housing situations. The purpose of this study was to better understand the contexts and service needs of unstably housed and homeless DV survivors, to promote more efficient and successful service matching from DV agencies. We examined whether DV survivors could be grouped by particular features, histories, and contextual factors, and how these group differences impacted what they needed from DV agencies. The sample included 406 homeless and unstably housed DV survivors who had recently sought DV services. Latent class analysis supported the identification of four distinct classes: (1) highest disadvantages service seeker, (2) moderate disadvantages—criminal legal system service seeker, (3) moderate disadvantages service seeker, and (4) lower disadvantages service seeker. Additionally, we were able to profile each class, and test the types of services survivors in each class needed from agencies.

## 1 INTRODUCTION

Domestic violence (DV) victim service programs in the United States share a commitment to providing survivor‐driven assistance (Cattaneo et al., 2020; Davies & Lyon, 2013), which means they focus on DV survivors' individual needs and circumstances rather than offering one set of predetermined services to everyone. This is important because each DV survivor is seeking something different from agencies: some are looking for legal assistance to end the relationship, some want emotional support more than tangible assistance, others need emergency respite from ongoing violence, and many are looking for a combination of supports and services. In short, survivors have a myriad of complex needs that require service programs to be flexible and comprehensive (Sullivan & Virden, 2017; Sullivan, 2011).

While the original intent of providing survivor‐driven services was based in the desire to be respectful of people's self‐determination and to counter abusers' controlling and isolating behaviors (Sullivan, 2018), this type of service provision has also been linked to positive outcomes for service‐seekers. DV survivors have reported finding agency assistance to be more helpful when they felt they had greater control over the services they received (Zweig & Burt, 2007). Further, survivor‐defined services have been linked to positive changes in survivors' sense of empowerment (Cattaneo et al., 2020).

In recent years, as affordable housing stock in the United States has continued to decline (Shaw, 2020), an increasing number of DV survivors are looking for long‐term housing assistance from programs, which is requiring staff to hone even more skills and to develop an even broader range of networks and resources to be maximally effective (Hernández‐Martinez et al., 2018; Stylianou & Pich, 2019; Sullivan et al., 2019). DV is a leading cause of homelessness and housing instability (Daoud et al., 2016; Pavao et al., 2007), and the economic abuse tactics used by perpetrators of DV can leave survivors with fewer economic resources to meet their housing needs in the already constrained U.S. housing market (Postmus et al., 2009). For example, in a study of 120 DV survivors, any economic abuse experienced predicted decreased economic self‐sufficiency (Postmus et al., 2009), which impeded survivors' ability to attain or sustain housing. Many abusive partners and ex‐partners intentionally sabotage survivors' efforts to sustain long‐term housing (Adams et al., 2018; Baker et al., 2010; Clough et al., 2014).

Given the prevalence of housing instability for DV survivors, it is critically important that DV agency staff identify, and respond to, the varied housing needs and housing barriers of their clients (Sullivan & Olsen, 2016). In line with service providers' commitment to providing survivor‐driven services (Cattaneo et al., 2020; Davies & Lyon, 2013), attention should be paid to a wide range of contextual factors that may need to be addressed to effectively assist survivors in obtaining safe and stable housing. Considering survivors' current situations (including current housing and financial status), potential housing barriers (such as criminal history and substance misuse), and protective factors (such as social support available), may provide a more holistic understanding of survivors' service needs, and support the provision of individualized services.

It may also be beneficial to explore which particular patterns of risk and protective factors suggest the need for different types and levels of service response. Some survivors, for example, require few resources and little time from staff to meet their needs (Sullivan et al., 2019). Others—especially those with a wider range of complex difficulties (Sullivan et al., 2019)—require far more of an agency's resources. For example, some DV survivors have well‐documented mental health needs, including high rates of posttraumatic stress disorder (PTSD), anxiety, severe depression, and suicide ideation, and services have been designed to achieve outcomes in those areas (Chandan et al., 2019; Sullivan, 2018). Having a better sense of how survivors' contexts and experiences coalesce to impact their service needs, as well as identifying the likely proportion of clients who may comprise each “category” of service need, may help service providers more effectively allocate their limited resources to best serve a wide range of survivors (Jahiel & Babor, 2007; Rog & Buckner, 2007). For example, one recent study, which involved surveying 577 homeless individuals about their service needs and barriers, used latent class analysis (LCA) to identify distinct groups of service seekers (Barile et al., 2020). As expected, people's service needs differed depending on the factors that contributed to their homelessness. Those who were homeless due exclusively to job loss—the largest grouping at 55% of the sample—also needed the fewest services. Those in the "disability/physical health class", on the other hand, were a much smaller group (4%) but comprised those needing the most services. While this study did not measure DV, and was comprised of primarily men, it illustrates the value of identifying factors related to service use.

Although few studies to date have examined the housing barriers and related service needs of DV survivors specifically, there is some evidence to suggest significant overlap between risk factors for DV and risk factors for homelessness. For example, people with disabilities are at increased risk for DV victimization (Breiding & Armour, 2015) as well as homelessness (Curtis et al., 2014). People of Color are also at greater risk than are their White counterparts for experiencing both DV (Breiding et al., 2014) and homelessness (Olivet et al., 2018). Other risk factors related to both DV and homelessness include substance misuse (Nilsson et al., 2019; Spencer et al., 2019) and having a criminal record (Iratzoqui & Cohn, 2020; Nilsson et al., 2019). Conversely, social support has been identified as a strong protective factor for both DV and homelessness (Dias et al., 2019; Johnstone et al., 2016; Phipps et al., 2019).

In areas like homelessness and DV, typologies of individuals in need of services can help guide practices or policies such that service matching would provide maximum benefit for the greatest number of clients (Rog & Buckner, 2007). It is important to understand not just the process and context of seeking services, but how that impacts the services received (Kennedy et al. 2012). DV is an area in which contextual factors *and* service needs are both important considerations in developing typologies that would support improved service delivery.

## 2 SCOPE OF THE STUDY

The current study was embedded within a larger, ongoing longitudinal demonstration evaluation aimed at examining the mechanisms through which mobile advocacy and flexible funding may lead to housing stability, safety, and well‐being for DV survivors over time. In the current investigation, we focused on exploring the experiences, contexts, and service needs of homeless and unstably housed survivors as they reached out to DV agencies for supportive services. The aims of this investigation were threefold: (1) examine whether unstably housed DV survivors can be grouped by housing and financial status, abuse experience, mental health and disability status, substance misuse, criminal history, and level of social support; (2) examine how group membership was associated with demographic variables such as race and ethnicity; and (3) examine how, if at all, subgroup differences impacted survivor needs from DV agencies.

## 3 METHODS

### 3.1 Participants

Over 400 homeless and unstably housed DV survivors are participating in a larger longitudinal study that began shortly after they sought services from one of five participating DV agencies in in the Pacific Northwest. Staff from each of the five agencies invited eligible clients to hear more about the research study. Eligibility criteria included (1) being a recent survivor of DV, (2) being homeless or at immediate risk of becoming homeless, (3) having sought services within the prior 3 weeks, and (4) speaking English or Spanish, or agreeing to participate with the assistance of an interpreter.

Our research team spoke with 438 eligible survivors who indicated an interest in hearing more about the study. Of those, 406 were willing and eligible to participate in interviews (93%). Survivors are being interviewed five times over the course of 24 months by a highly trained interviewer, with interviews spaced every 6 months. Participants are paid $50 for each interview. All procedures were approved by Michigan State University's institutional review board.

### 3.2 Procedures

The current study utilized the data collected at baseline, which includes demographics, historical data regarding abuse and homelessness, as well as information regarding survivors' contextual and service needs. The final sample of 406 participants consisted of predominantly cisgender female (97%) and heterosexual (86%) survivors of DV. Ages ranged from 19 to 62, with an average of 34.5 years old. Thirty‐five percent of survivors self‐identified as Non‐Hispanic White, and 65% reported an ethnic/racial minority: Hispanic/Latinx (35%), Black (19%), US Indigenous (12%), Asian (4%), and/or Middle Eastern (1%). Of survivors who reported an ethnic‐minority identity, 15% selected more than one race/ethnicity category, indicating multiracial or multiethno‐racial identities. Most survivors identified English as their primary language (80%). Immigrant survivors represented 18% of the sample. Participants' highest level of education varied considerably: 29% had not completed high school, 22% had a high school diploma/GED, 36% had some vocational training or had attended college classes, and 13% had either Associate's, Bachelor's, or Advanced degrees. Most study participants (73%) had a prior history of homelessness and the vast majority of the sample (87%) had stayed with family or friends at least once to avoid homelessness. For more detailed demographic data see Table 1.

**Table 1:** Sociodemographics of sample (*n*=406)

	*n*	*%*
Gender		
Female	393	96.8
Male	9	2.22
Gender queer/nonconforming	4	0.99
Race/Ethnicity		
Non‐Hispanic White only	144	35
Multiracial/multiethnic	62	15
Hispanic/Latinx	142	35
Black	76	19
Asian	16	4
US Indigenous	48	12
Middle Eastern	5	1
Sexual Orientation (*N* = 405)		
LQBQA	55	13.55
Heterosexual	350	86.31
Children		
Yes	299	73.65
Number of minor children parenting/responsible for		
1 child	127	31.28
2–3 children	136	33.5
4 or more children	36	8.87
Previous Employment Status		
Employed in the last 6 months	235	57.88
Current Employment Status (*N* = 405)		
Employed, working 41 or more hours per week	28	6.91
Employed, working 30–40 h per week	52	12.84
Employed, working less than 30 h per week	58	14.32
Employed seasonally	5	1.24
Not employed	259	63.95
Disabled, not able to work	3	0.74
Education		
8th grade or less	40	9.85
Between 9th–12th grade	77	18.97
High school graduate	49	12.07
GED	40	9.85
Vocational school/training certificate	33	8.13
Some college	86	21.18
Associate degree	28	6.9
Bachelor's degree	35	8.62
Advanced degree	18	4.43
Immigration status		
US citizen	331	81.53
Permanent resident	19	4.68
Not US citizen or permanent resident	56	13.79

Abbreviation: LQBQA: lesbian, gay, bisexual, or queer.

### 3.3 Measures

Interviews averaged about 75 min and covered a wide variety of topics including demographics, employment status, well‐being, mental health, history of abuse, housing, income, schooling and child behavior, and service needs. The following measure descriptions are only those used for the current analysis. For several measures, we constructed measurement models to measure the constructs and outputted the factor scores as variables in the LCA models. The primary benefit of factor scores over raw sum or mean scores is that factor scores do not include measurement error and therefore provide an unbiased measure of the constructs. Additionally, in generating factor scores, we can relax the assumption that measurement items carry similar weights (Brown, 2015; Curran et al., 2014).

### 3.4 Latent class model variables

#### 3.4.1 Current homelessness status

Participants reported their current housing location (e.g., a transitional housing, at a friend or relative's house or apartment and paying part of the rent). Responses were recategorized to indicate participants who reported being homeless or not. Following the definition provided by the U.S. Department of Housing and Urban Development (HUD) (HUD, 2013), homelessness was operationalized as lacking a fixed, regular, and adequate nighttime residence. Within this sample, that included people currently staying in hotels, anywhere outside, in vehicles, abandoned buildings, or in DV or homeless shelters.

#### 3.4.2 Barriers to obtaining housing

Participants responded to questions about common barriers faced when seeking housing using a modified version of the 19‐item index by Gubits et al. (2015) (e.g., poor or no credit history, transportation issues, history of eviction) and four additional items generated for this study (e.g., owing back rent, having unpaid utility debt). Results of an exploratory factor analysis (EFA) with a calibration sample (*n* = ~122) and a confirmatory factor analysis (CFA) with a validation sample (*n* = ~284) produced a 5‐factor model with good model fit (*CFI* = 0.93; *TLI *= 0.92; *RMSEA *= 0.046). The five factors were (1) Housing barriers around finances (e.g., paying rent, security deposit; 3 items), (2) Housing barriers around rental history (e.g., no credit history, no references; 5 items), (3) Legal housing barriers (e.g., felony convictions, discrimination; 4 items), (4) Negative housing events (e.g., history of eviction, lease violations issues with past landlords; 5 items), and (5) Housing barriers related to dependents (e.g., having children in the house, caring for someone with a disability; 4 items). The *α* coefficient for the scale was 0.78 (*M* = 1.98, *SD* = 1.48).

#### 3.4.3 Number of moves

Participants indicated the total number of times they had moved in the last 6 months. We recoded this variable into (0) no moves, (1) one to two moves, (2) three to four moves, (3) five to six moves, (4) seven to eight moves, (5) more than 8 moves.

#### 3.4.4 Intimate Partner Violence

Experience of IPV through physical abuse, emotional abuse, sexual abuse, and stalking or harassment were measured using a modified version of the Composite Abuse Scale (CAS) (Hegarty et al., 1999; Loxton et al., 2013). Five new items were added to the scale to better capture stalking, strangulation, and sexual assault. Participants responded to items using a scale ranging from 0 = “Never” to 5 = “Daily.” Cronbach's *α* for the full measure was 0.95 (*M* = 1.69, *SD* = 1.53). Separately, to measure abuser's use of economic restriction and exploitation we used the Revised Scale of Economic Abuse (SEA2; Adams et al., 2020). Sample items included asking how often in the prior six months the abuser would “force or pressure you to give them your savings or other assets,” and “keep you from having a job or going to work.” Response options ranged from 0 = “never” to 4 = “quite often.” Cronbach's alpha for the measure was .91 (*M = 1.46, SD = 1.05)*.

#### 3.4.5 Mental health

Participants were asked to respond to the 10‐item Trauma Screening Questionnaire (TSQ; Kroenke et al., 2001), 9‐item Patient Health Questionnaire (PHQ‐9; Brewin et al., 2002), and 7‐item Generalized Anxiety Disorder measure (GAD‐7; Spitzer et al., 2006) to assess PTSD, depression, and anxiety, respectively. Using scale cut‐offs, a variable was created in a yes/no format to depict participants whose scores indicated the existence of PTSD, as well as severe symptoms for depression and anxiety.

#### 3.4.6 Financial instability

Participants responded to ten questions about their finances using the Adequacy of Financial Support scale, modified from Mowbray et al. (1999). Items assessed whether they had enough money in the prior 6 months for life expenses (e.g., food, rent/mortgage, utilities, childcare, and transportation) using a 4‐point scale of difficulty (0 = “Not difficult at all” to 3 = “Very difficult”). For analysis, a mean scale score was created. Cronbach's *α* for the 10‐item measure was 0.87 (*M* = 2.28, *SD* = 0.68).

#### 3.4.7 Unemployment status

In a yes/no format, participants were asked if they held any employment in the past six months. We reverse coded this item for the analysis.

#### 3.4.8 Criminal record

In a yes/no format, participants were asked if they had a criminal charge that would show up in a background check.

#### 3.4.9 Physical disability

In a yes/no format, participants were asked if they have a physical disability or disabling condition.

#### 3.4.10 Social support

The 6‐item Medical Outcomes Study Social Support Survey (MOS‐SSS‐6; Holden et al., 2014) was used to measure social support. Items assessed how confident participants felt about others in their lives that could support them in times of need (e.g., “How much of the time would you say you currently have someone in your life who you could do something enjoyable with?*”*) using a 5‐point Likert scale (i.e., “None of the time” to “All of the time”). For analysis, a mean scale score was created. Cronbach's *α* was 0.90 (*M* = 3.28, *SD* = 1.15).

#### 3.4.11 Substance misuse

The widely used CAGE–AID (Adapted to Include Drugs) tool was used to assess substance misuse (Ewing, 1984). Response options are yes/no (0 = no and 1 = yes). The original tool includes four questions necessary to ascertain alcohol and illicit drugs use such as *“*Have you ever felt you ought to cut down on your drinking or drug use?*”* The items were modified for the current study to include eight items—four questions assessing drug use and four questions assessing alcohol use. Cronbach's *α* for the full measure was 0.75. For each of the subscales (four items measuring alcohol use and four items measuring drug use), two or more positive answers are considered an indication of abuse. Using these thresholds, we created dichotomous variables for indication of abuse (1) and no indication of abuse (0) for both drugs and alcohol.

### 3.5 Class profiling variables

#### 3.5.1 Demographics

Participants provided a number of sociodemographic characteristics including race, ethnicity, education, parental status, and immigration status. For the purposes of this analysis, we recoded race to be a dichotomous variable where 1 signifies participants with one or more minority racial identity and 0 signifies single race White participants. For ethnicity, Hispanic/Latinx participants were coded as 1 and non‐Hispanic/Latinx participants as 0. Education was collapsed into two groups of participants who had completed high school or more schooling and those who completed less than a high school degree. For parental status, if a participant was caring for a child under the age of 18, they received a 1. For immigration status, participants with US citizenship were coded as 1.

#### 3.5.2 History of homelessness

Participants reported the number of times they had been homeless in their lifetime. Five or more times homeless was collapsed into one category. We also captured information about whether participants had been homeless before the age of 18 in a yes/no format.

### 3.6 Distal outcomes

#### 3.6.1 Service needs

Participants responded to 14 yes/no questions about the services they hoped to receive from the agency. These services were housing, employment, education, financial help, legal assistance, childcare, counseling, transportation, healthcare, issues for children (besides childcare), food, clothing, other material goods/services, and increasing social support. We created a sum score of these items for a *Total Number of Needs* variable (*α* = 0.70). We also conducted an EFA and CFA using these 14 items. The model fit the data well using a 4‐factor structure (*CFI* = 0.92; *TLI* = 0.89; RMSEA = 0.043). The four factors were (1) vocational and transportation needs (three items: employment, education, and transportation); (2) financial and housing needs (five items: housing, financial help, food, clothing, material goods); (3) child‐related needs (two items: childcare, issues for children); and (4) specialized needs (four items: counseling, legal assistance, healthcare, and social support). Factor scores for each factor were calculated and used in subsequent analyses.

### 3.7 Analytic procedures

#### 3.7.1 Latent class analysis

We used LCA with a combination of categorical and continuous indicators to identify unobserved groups or classes of DV survivors who are similar based on participants' housing and financial status, abuse experience, mental health and disability status, substance misuse, criminal history, and level of social support.

##### Missing data

Only 1.5% of cases had missing data (*n* = 6) on the LCA indicator or outcome variables, and these cases were removed using listwise deletion.

##### Variable selection

Estimating too many parameters in an LCA model will result in instability of the model. Therefore, to exclude excessive variables, we conducted a series of variable selection analyses. The variable section model is a four‐equation model, in which models were simultaneously estimated using maximum likelihood (ML) as the estimator in Mplus version 8.5 (Muthén & Muthén, 2020). The needs of survivors were categorized into four dependent variables for variable selection. Model specification:

(1) $$\mathrm{Vocational}\;\mathrm{and}\;\mathrm{Transportation}\;\mathrm{Needs}=B^{1}Χ+E^{1},$$

(2) $$\mathrm{Financial}\;\mathrm{and}\;\mathrm{Housing}\;\mathrm{Needs}=B^{2}Χ+E^{2},$$

(3) $$\mathrm{Specialized}\;\mathrm{Needs}=B^{3}Χ+E^{3},$$

(4) $$\mathrm{Child}-\mathrm{related}\;\mathrm{Needs}=B^{4}Χ+E^{4},$$

where, ${В}^{1}$, ${В}^{2}$, ${В}^{3}$, and ${В}^{4}$ are the vectors of the regression coefficients. The $E^{1}$, $E^{2}$, $E^{3},$ and $E^{4}$ are the error terms of the regression models. To begin the variable selection process, we used a comprehensive set of explanatory variables that conceptually could be related to DV survivors' needs. Next, we employed backward selection and calculated the partial *r* ^2^ explained by a new explanatory variable each time we ran the regression models. We dropped the explanatory variables that provided least partial *r*‐squared. The variables selection process stopped when we observed a large drop of the total *r* ^2^. Only the variables that were retained are presented in the measures section.

##### Research aim 1: Latent class enumeration

To determine the appropriate number of latent classes, we followed class enumeration procedures, wherein we began by specifying an LCA model with one class and evaluated the following fit statistics: Bayesian Information Criterion (BIC; Schwarz, 1978), the Akaike's Information Criterion (AIC; Akaike, 1974), the sample‐size adjusted Bayesian information criterion (aBIC; Sclove, 1987); the Vuong–Lo–Mendell–Rubin likelihood ratio test (VLMR; Lo et al., 2001), and the adjusted Lo‐Mendell‐Rubin likelihood ratio test (aLMR; Sclove, 1987). We repeated this process with each subsequent class model. For each additional model, lower values on AIC, BIC and aBIC indicated superior model fit as compared with the prior model. The aLMR and VLMR were used to assess model fit, such that statistical significance on the aLMR and VLMR likelihood ratio tests indicated whether the model has significantly improved from the prior model. The final step of the class enumeration process included assessing for interpretability, size, and uniqueness of the latent classes.

##### Research aim 2: Profiling class membership

To address the second research aim, we used the automatic R3step command in Mplus version 8.5 (Asparouhov & Muthén, 2014; Vermunt, 2010). The R3step command is a three‐step method using a multinomial logistic regression approach for determining if there are significant predictors of class membership. This method takes the results of the LCA (uninfluenced by auxiliary predictor variables: first step) and estimates most likely class membership (second step). Finally, the model uses a multinomial logistic regression to regress class membership on the auxiliary predictor variables to evaluate their relationships with the class solutions while taking into account misclassification during the second step (third step). Our auxiliary predictor variables included race, ethnicity, citizenship, parental status, education, and history of homelessness.

##### Research aim 3: Testing distal outcomes

In the final step of the analysis, we employed the automatic DU3step function in Mplus version 8.5 (Muthén & Muthén, 2020) to test whether the emergent classes were associated with survivors' needs from DV agencies. Like the R3step function, the DU3step takes the results of the LCA (uninfluenced by auxiliary distal outcome variables: first step) and estimates most likely class membership (second step). The outcome variables are treated as distal variables assuming unequal means and variances in each class (Asparouhov & Muthén, 2014). Finally, the third step uses a maximum likelihood methods and χ^2^ equality of means tests to assess for differences in service needs across classes (Asparouhov & Muthén, 2014; Vermunt, 2010).

## 4 RESULTS

### 4.1 Description of the sample

At study entry, 42% of the participants were homeless (36% living in a shelter, and 6% unsheltered homeless). The other 58% of participants were unstably housed: 24% were in homes they owned or were renting, 22% were staying with family and friends without paying rent, 9% were living with family and friends and paying part of the rent, and 3% were in transitional housing or a drug treatment program.

Most study participants (73%) had a prior history of homelessness. Of those who had been homeless, the average cumulative amount of time spent homeless was just over 2 years. Almost a third of those with a history of homelessness had been homeless at least once before age 18. Seventeen percent of all participants had been in foster care. The vast majority of the sample (87%) had stayed with family or friends at least once to avoid homelessness.

Over half of the participants had been employed (58%) at some point in the 6 months before participating in the study, but only 35% were employed at study entry. Of those who had lost their jobs in the prior 6 months, 70% reported it was due to the abuse they had experienced.

Not surprisingly, participants had experienced a range of DV in the prior 6 months. Forms of abuse included emotional (96%), physical (93%), stalking/harassment (90%), economic (89%), and sexual (53%). On average, participants reported moderate depression (*M* = 12.99, *SD* = 6.37) and moderate anxiety (*M* = 12.16, *SD* = 6.28). With regard to post traumatic stress disorder (PTSD), a score of six on the TSQ signifies the presence of PTSD and the average score was just above this cutoff (*M* = 6.88, *SD* = 2.48).

### 4.2 Research aim 1: Latent class enumeration

**Figure 1:**
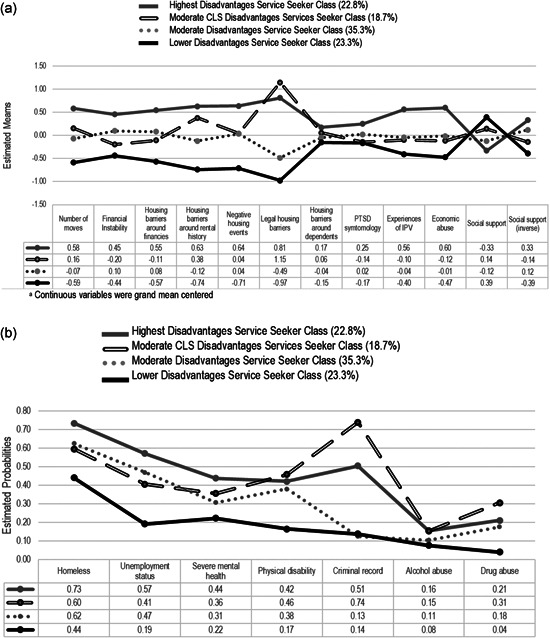
(a) Response patterns and item probability plots for continuous variables. (b) Response patterns and item probability plots for categorical variables

Our first research aim explored whether we could identify distinct classes of DV survivors based on similar characteristics and contextual barriers. Following class enumeration procedures, we generated LCA models with solutions ranging from one to six classes to identify the best fitting model. As shown in Table 2, examination of the AIC, BIC, aBIC, VLMR, and aLMR supported identification of the four‐class model. Although the three‐class model showed potential (lower AIC, BIC, ABIC), the significant aLMR indicated that adding a class leads to statistically significant improvement in model fit. Furthermore, the four‐class solution breaks out the largest class into two more interpretable classes while retaining adequate class sizes (>5% of data). Figure 1a illustrates the response patterns on the continuous variables for the four classes. Figure 1b presents the item probability plots for the categorical variables. As shown, the classes were clearly distinguishable on all variables entered into the model. The four‐class solution illustrates a clear high, medium, and low placement of the class assignments, with the middle group breaking out into two classes.

**Table 2:** Goodness‐of‐fit indicators for latent class model

	AIC	BIC	aBIC	aLMR	VLMR	Entropy
1 Class	14396.11	14519.93	14421.56	–	–	–
2 Classes	11643.12	11834.83	11682.52	0.00	0.00	0.85
3 Classes	11458.41	11726.01	11513.41	0.08	0.08	0.83
4 Classes	11298.07	11641.55	11368.67	0.04	0.04	0.87
5 Classes	11159.29	11578.65	11245.48	0.50	0.50	0.88
6 Classes	11067.45	11562.70	11169.24	0.22	0.22	0.88

Abbreviations: AIC, Akaike's Information Criterion; BIC, Bayesian Information Criterion; aBIC, adjusted Bayesian information criterion; aLMR, adjusted Lo‐Mendell‐Rubin likelihood ratio; VLMR, Vuong–Lo–Mendell–Rubin likelihood ratio.

We labeled Class (1) *highest disadvantages service seeker* and Class (4) *lower disadvantages service seeker. Lower disadvantages service seekers* had the lowest scores on all class indicators. In contrast, participants assigned to the *highest disadvantages service seeker* class reported higher scores on all class indicators. Two distinct classes comprised the moderate disadvantage service seekers. Participants in these two classes had similar scores between the lower and highest disadvantage service seeker classes across almost all indicators, with three exceptions. What distinguished these two moderate classes were the scores on criminal legal system (CLS) variables (e.g., legal housing barriers, criminal record, and drug abuse). As a result, we labeled Class (2) *moderate disadvantages CLS service seeker*, and Class (3) *moderate disadvantages service seeker*. The *moderate CLS* class had the highest scores across all four classes on the three criminal‐legal focused variables. In addition, participants assigned to the *moderate CLS* class had slightly higher item response probability of having a physical disability than the *highest disadvantages* class.

As illustrated in Figure 1a,b, participants assigned to the *moderate disadvantages service seeker* class comprised the majority of participants (35.3% of the sample; *n *= 143). The *lower disadvantages service seeker* class comprised 23.3% of participants (*n* = 94). The *highest disadvantages service seeker* class compromised 22.8% of participants (*n* = 91). Finally, the smallest class was the moderate disadvantages CLS service seeker class, which comprised 18.7% of the sample (*n* = 73). Three indicators—housing barriers related to children, current PTSD symptomology, and alcohol abuse—were not adequate differentiators of class membership.

### 4.3 Research aim 2: Profiling latent classes

Our second research aim focused on understanding whether class compositions differed demographically or based on survivors' homelessness histories. Multinomial logistic regression results are presented in Table 3. As illustrated, the four classes did not significantly differ based on race, ethnicity, citizenship, education, or parental status. The only significant differences were based on participants' homelessness histories. Individuals assigned to the *highest disadvantages service seeker* class had greater history of homelessness (*M* = 2.86, *SD*  = 1.95), followed by *the moderate CLS class* (*M* = 2.83, *SD* = 1.82), then the *moderate disadvantages service seeker class* (*M* = 1.83, *SD* = 1.76), and finally, the *lower disadvantages services seeker class* (*M* = 1.41, *SD* = 1.67). The differences among these groups were significant when comparing *the highest disadvantages class* to the *moderate disadvantages* class (*b* = −0.28, *p* = 0.001) and *lower disadvantages* class (*b *= −0.50, *p* < 0.001). There was no significant difference between the *moderate CLS class* and the *highest disadvantage class* (*b* = 0.11, *p* =  0.29) in history of homelessness. Individuals in both the *moderate disadvantages* class and the *moderate CLS class* had a significantly greater history of homelessness compared with the *lower disadvantages class* (*b* = −0.22, *p* = 0.04; *b *= −0.40, *p* < 0.001). Although it was not statistically significant, results suggest that non‐US citizens were less likely to be assigned to the *moderate CLS disadvantage class* when compared to all other classes.

**Table 3:** Multinomial logistic regression coefficients for the 4‐Class model with covariates

	Effect	*b*	*SE*	Odds ratio
**Highest disadvantages service seeker** vs. lower disadvantages service seeker class	Race	0.28	0.40	1.32
	Ethnicity	0.30	0.47	1.35
	Citizenship	−0.47	0.51	0.63
	Parent	−0.30	0.37	0.74
	Education	−0.60	0.39	0.55
	Homelessness History	0.50***	0.12	1.65
**Highest disadvantages service seeker** vs. moderate disadvantages service seeker class	Race	−0.16	0.36	0.85
	Ethnicity	0.14	0.49	1.15
	Citizenship	0.37	0.48	1.44
	Parent	−0.46	0.35	0.63
	Education	0.07	0.38	1.08
	Homelessness History	0.28**	0.89	1.33
**Highest disadvantages service seeker** vs. moderate CLS disadvantages service seeker class	Race	0.25	0.40	1.29
	Ethnicity	0.67	0.59	1.95
	Citizenship	−2.58	1.89	0.08
	Parent	−0.22	0.39	0.80
	Education	−0.38	0.43	0.69
	Homelessness History	0.11	0.10	1.11
**Moderate CLS disadvantages service seeker** vs. moderate disadvantages service seeker class	Race	−0.41	0.38	0.66
	Ethnicity	−0.53	0.57	0.59
	Citizenship	2.95	1.85	19.08
	Parent	−0.24	0.39	0.79
	Education	0.45	0.42	1.57
	Homelessness History	0.18	0.09	1.19
**Moderate CLS disadvantages service seeker** vs. lower disadvantages service seeker class	Race	0.02	0.41	1.03
	Ethnicity	−0.37	0.55	0.69
	Citizenship	2.11	1.83	8.26
	Parent	−0.08	0.40	0.93
	Education	−0.23	0.42	0.80
	Homelessness History	0.40***	0.11	1.49
**Moderate disadvantages service seeker** vs. lower disadvantages service seeker class	Race	0.44	0.39	1.55
	Ethnicity	0.16	0.42	1.17
	Citizenship	−0.84	0.42	0.43
	Parent	0.16	0.37	1.17
	Education	−0.68	0.37	0.51
	Homelessness History	0.22*	0.11	1.25

Abbreviation: CLS, criminal legal system.

* *p* < 0.05

** *p* < 0.01

*** *p* < 0.001.

### 4.4 Research aim 3: Testing distal outcome

Our final aim tested whether the four distinct classes of survivors could predict the number and types of resources and services that survivors seek out from DV agencies. These results are presented in Table 4. First, we tested whether the latent classes could predict the number of resources and services survivors reported needing from the agency. The mean number of services survivors hoped to receive differed significantly across classes. The l*ower disadvantages service seeker* class had a significantly fewer number of needs (*M* = 7.81) than all other classes. The *highest disadvantages service seeker class* wanted a significantly higher number of services than all other classes (*M* = 10.82), except the *moderate disadvantage* class (*M* =  9.88), which was not statistically significant. The *moderate CLS disadvantage class* (*M*  = 8.95) had significantly fewer needs than the *moderate disadvantage class*.

**Table 4:** Results of equality tests of means for 4‐Class model with distal outcome variables

	Lower disadvantages service seeker class		Moderate disadvantages service seeker class		Moderate CLS disadvantages service seeker class		Highest disadvantages service seeker class	
	*M*	*SE*	*M*	*SE*	*M*	*SE*	*M*	*SE*
Number of needs	7.81	0.30	9.88	0.24	8.95	0.38	10.82	0.38
Vocational and transportation needs	−0.91	0.06	−0.36	0.05	−0.37	0.07	−0.03	0.07
Financial and housing needs	−0.28	0.04	0.14	0.03	0.04	0.05	0.41	0.04
Child‐related needs	0.71	0.09	0.97	0.07	0.79	0.10	1.04	0.09
Specialized needs	−0.80	0.05	−0.48	0.04	−0.75	0.05	−0.33	0.05

	Equality tests of means							
	*χ* ^2^ highest disadvantages vs. lower disadvantages	*χ* ^2^ highest disadvantages vs. moderate disadvantages	*χ* ^2^highest disadvantages vs. moderate CLS disadvantages	*χ* ^2^ moderate CLS disadvantages vs. moderate disadvantages	*χ* ^2^ moderate CLS disadvantages vs. lower disadvantages		*χ* ^2^moderate disadvantages vs. lower disadvantages	
Number of needs	40.12***	3.77	9.07*	4.36**	5.47*		26.51***	
Vocational and transportation needs	85.38***	12.47***	10.89**	0.04	32.32***		39.47***	
Financial and housing needs	126.06***	21.48***	24.35***	2.40	23.13***		59.61***	
Child‐related Needs	7.10**	0.33	3.16	1.92	0.38		4.62*	
Specialized Needs	54.81***	6.34*	39.10***	18.11***	0.62		27.40***	

*Note*: Reported means are based on the residuals from regression models that account for covariates.

Abbreviation: CLS, criminal legal system.

* *p* < 0.001

** *p* < 0.05

*** *p* < 0.001.

Second, we tested whether the types of resources and services could be predicted by the latent classes. Using the four factor scores: (1) vocational and transportation needs; (2) financial and housing needs; (3) child‐related needs; and (4) specialized service needs, we tested the equality of means across classes and were able to significantly predict the type of resources and services survivors in each class needed from the agency. Survivors in the *highest disadvantages service seeker* class had significantly higher scores in all four DV service provision categories compared with the *lower disadvantages service seekers*. Both *moderate disadvantages service seeker* classes had significantly higher scores than the *lower disadvantages service seeker* class in all domains. Across the two moderate disadvantages classes, the moderate CLS class needed significantly more services related to specialized needs (e.g., counseling, legal assistance, healthcare, and social support) compared with the moderate disadvantages class.

## 5 DISCUSSION

The results of this study complement several others in emphasizing the importance of matching interventions and services to the unique needs of individuals seeking help (Goodman et al., 2016; Nichols, 2013; Sullivan, 2018). Individuals who have recently experienced DV commonly report needing a range of assistance to ensure their safety and well‐being (Sullivan & Virden, 2017; Thomas et al., 2020). In this study we found that, among homeless or unstably housed DV survivors, there were four distinct types of service seekers, distinguished by the number of difficulties and disadvantages they brought with them when seeking help. Interestingly, groups did not differ by type of disadvantage, but by severity. Furthermore, the four groups did not differ by race, ethnicity, citizenship, education, or parental status. The class differences in health, social support, DV victimization, financial and housing instability, and criminal history were consistently in magnitude only.

Evidence for the link between level of disadvantage and need for services was apparent in our findings. Each class was significantly associated with both the number and type of services survivors reported needing from the agency, such that those with the highest disadvantages reported wanting a significantly higher number and broader range of services from the agency compared with the other classes. These findings support the deduction that there are diverse subgroups of survivors seeking services and these groups may want and need different types and levels of assistance (Cattaneo et al., 2020; Davies & Lyon, 2013; Goodman et al., 2016). These findings also corroborate the importance of providing survivor‐centered advocacy to assist survivors in obtaining safe and stable housing. Having a clear sense of survivors' contextual realities, including specific disadvantages and potential barriers to housing, may provide important insight to effectively respond to survivors' unique needs and goals.

Notably, although survivors within the four classes differed in the number and types of services needed, all four still hoped for significant support. The mean number of resources and services requested from survivors in the lowest need group was over seven (out of 14), highlighting that even DV survivors with fewer disadvantages still require significant assistance to obtain safe and stable housing. Furthermore, this finding points to the importance of strong collaborations between DV agencies and other community resources to best support the needs of survivors and their families, suggesting that it is critical for advocates to be knowledgeable of other supportive services and resources available in their communities. This corroborates other findings that suggest that connecting survivors to the multiple services they need may be essential for survivors' housing stability, particularly those with legal barriers (Kubiak et al., 2011; Sullivan, López‐Zerón et al., 2019).

The types of disadvantages measured in this study support and advance what we know about common housing barriers facing DV survivors (Adams et al., 2018; Daoud et al., 2016). In particular, our findings suggest that survivors in the *highest disadvantages* class, who were the most likely to be homeless and unemployed upon entry into services, also needed an array of additional services, including mental health services, transportation, and other safety‐related services, alongside traditional housing services. This is an important finding as it highlights how critical it is to tailor services to survivors' unique needs. It is clear that although some needs might not be typically associated with housing stability (e.g., transportation and employment), they may impact survivors' ability to obtain and maintain safe and stable housing.

The *moderate CLS disadvantages* class members were more likely to have legal‐related disadvantages, including having a criminal history, drug use, and other legal‐ related housing barriers. Housing scholars have consistently found evidence that possessing a criminal history exacerbates the process of obtaining safe and stable housing and employment (Barile et al., 2018; Evans et al., 2019; Jason et al., 2007; Purtle et al., 2020). However, although DV survivors may share some similar barriers to accessing employment and housing as other unstably housed populations, the impact of prior criminal histories is less understood in this population. Regardless of whether a survivor's criminal history is related to DV, it can drastically impact housing and employment access (Kubiak et al., 2011). As such, this finding has important implications for the training of advocates to effectively address survivors' criminal background. This may include expunging prior records, understanding survivors' housing rights related to felonies and misdemeanors, and advocating with landlords around this issue (Kubiak et al., 2011).

In addition to requiring a high number of services from agencies, participants assigned to the *moderate CLS class* were also significantly more likely to report needing specialized services from DV agencies. In this study, specialized services were operationalized as services other than the “typical” advocacy services provided at DV agencies. This included counseling, legal assistance, health care, and social support. This finding has important implications for the allocation of DV service and advocacy funding to support navigation of legal and physical and mental health systems. In addition, providing space and resources to bolster social support is something that DV service providers try to do when resources allow, and these findings reinforce recommendations to promote ongoing counseling or social support groups, for example (Guyon‐Harris et al., 2017).

Another key difference between the classes was the presence of a physical disability. Individuals in the *moderate CLS disadvantages*, the *highest disadvantages*, and the *moderate disadvantages* classes were more likely to report the presence of a physical disability compared with the *lower disadvantages* class. Barile and colleague's LCA examining pathways to homelessness similarly found that individuals with physical disabilities reported higher needs and differential barriers to accessing services, particularly around transportation (Barile et al., 2018, 2020). As such, our findings join others in highlighting the importance of considering clients' disabilities when providing services. While DV advocates are often aware of the mental health issues that come with experiences with DV, less is known about how that is compounded by living with a disability while unstably housed. Future research should consider investigating such overlapping disadvantages particularly in relation to housing‐related help seeking.

Class membership also differed by level of drug misuse and abuse, with those in the *highest disadvantage* groups most likely to report this behavior. This finding corroborates other studies evidencing a link between DV victimization and substance abuse. For example, a national survey found that DV victims were significantly more likely to actively use cannabis, cocaine, and opioid use, or experience problems related to these substances than those who had not experienced DV (Smith et al., 2012). Another study found that women attending a methadone clinic were three times more likely to report frequent heroin use if they had also reported DV (El‐Bassel et al., 2005). Given the varied detrimental impacts substance abuse can have on a DV survivors' health, housing, employment and well‐being, findings support the efforts that DV programs are making with substance abuse treatment programs to effectively serve this population (Macy & Goodbourn, 2012).

In line with prior research on the importance of social connection, participants in the class noting *lower disadvantages* also reported having higher levels of social support, and vice versa with *the highest disadvantage* class (Liang et al., 2005). Some DV programs have intentionally created services and responses around improving survivors' social support, with some evidence that these actions lead to their improved well‐being (Ogbe et al., 2020). This finding is also consistent with a growing body of literature suggesting that survivors prefer DV agencies, shelters, and housing solutions that promote community building and social support (Hetling et al., 2020). Goodman and Smyth (2011) have specifically called for taking a social network‐oriented approach with survivors, involving their informal support networks in their safety and recovery. Given the number of people in this study who were staying with family or friends or who had done so in the past to avoid homelessness, this approach seems especially important in preventing homelessness among DV survivors.

### 5.1 Limitations

Findings need to be considered in light of study limitations. As with any exploratory analysis, there is always a possibility that relevant themes may have been misinterpreted, overlooked, or over emphasized. First, we may have unintentionally excluded variables that should have been considered for the LCA model. Given the litany of factors that could influence DV service needs, we relied on theory and a statistical variable selection process to determine which variables to include in the model. As such, several of the variables included in this analysis are factor scores. Although this allowed us to include a wider variety of topics in the LCA model, we simultaneously lost information about individual items. Nonetheless, we remain confident in the classes observed and the rigorous methods applied to our variable selection process. Additionally, these data are cross sectional baseline data and therefore represent participants who have not yet received services from the agency. Thus, this study is limited by only relying on reports of what participants needed *before* receiving services. We are aware that service need is not a time invariant variable and are interested in exploring how service needs change over time for these classes of DV survivors. However, the data for subsequent timepoints are still being collected. Moving forward, we hope to build on this analysis to look at whether the classes found in this study change over time and the extent to which different survivors' needs are met.

The generalizability of findings may be impacted by a number of factors. All of the study participants were not only DV survivors but also unstably housed or homeless at the start of the study. Most identified as heterosexual and cisgender. While the sample was heterogeneous in terms of race and ethnicity, it was primarily Latinx, African American or White, and more studies are needed with a higher number of Native American and Asian American participants. Finally, this study was conducted in the Pacific Northwest, and may reflect some contexts and service needs specific to that geographic area.

### 5.2 Policy and practice implications

There are several important implications to glean from this study for both policy and practice. This study joins a growing evidence base demonstrating the utility of identifying distinct groups of service seekers who may subsequently require different services (e.g., Barile et al., 2020; Bridges et al., 2012; Jahiel & Babor, 2007; Theodos et al., 2012). While such classes should never be used to drive or mandate services, they can be useful in helping practitioners and policy makers embrace the need for providing clients with a wide range of assistance. This requires planning for and allocating agency resources (e.g., staffing, training, and time) so that those clients with fewer needs do not receive assistance they do not request or want, while clients with greater and interrelated disadvantages receive the additional resources and help they are seeking. This may mean that a small but important group of clients should be expected to need a wider array of services for a longer period of time. While resource‐intensive, if those with fewer or less intensive needs are not provided services they are not seeking, agencies can ideally balance resources accordingly (Culhane et al., 2011).

Uncovering evidence for multiple types of service seekers also has important implications for the types and level of services DV agencies provide. For example, advocates may be able to assess whether a client needs a “light touch” of service provision as opposed to a high‐needs client that requires a heavier dose of service provision, and adapt their caseloads accordingly. Furthermore, having a concrete understanding of the various experiences, contextual realities, and potential housing barriers of unstably housed survivors could significantly help advocates prioritize their efforts accordingly. This holistic understanding may provide advocates with the necessary insight to adequately tailor services to effectively respond to survivors' individual needs.

Nevertheless, DV advocates working on the front lines might find evidence of higher, moderate, and lower need classes of survivors unsurprising, as advocates see a diversity of survivors and their stories each day. Advocates may already be able to determine survivor needs at intake but are limited by the resources available to adequately address survivor needs. As such, these findings might be best disseminated to policy makers and institutions that fund such DV services to account for extra training, staff, resources, and funding time required to provide adequate support around housing for multiply disadvantaged DV survivors. Additionally, these findings speak to the importance of community mobilizing and collaborations across agencies on the front lines. With the ever‐shifting landscape of housing equity in the United States, we urgently need collaborative synergy across communities in efforts to safely house disadvantaged community members.

### 5.3 Conclusion

This study aimed to gain a broader understanding of the range of contextual factors influencing the service needs of homeless and unstably housed DV survivors. Overall, findings from this analysis elucidate the diversity in housing needs and barriers faced by DV survivors as well as highlight critical aspects of providing adequate and survivor‐centered services when working with unstably housed and homeless DV survivors. The results further reveal important implications for the ways in which DV agencies can promote efficient service matching. Finally, findings add to the burgeoning literature around attending to the multitude of factors that influence homelessness prevention for DV survivors.

## CONFLICT OF INTERESTS

The authors declare that there are no conflict of interests.

## Data Availability

Author elects to not share data as data collection is ongoing. After the study ends, data will be made available.

## References

[jcop22637-bib-0075] Adams, A. E. , Greeson, M. R. , Littwin, A. K. , & Javorka, M. (2020). The Revised Scale of Economic Abuse (SEA2): Development and initial psychometric testing of an updated measure of economic abuse in intimate relationships. Psychology of Violence, 10(3), 268–278. 10.1037/vio0000244

[jcop22637-bib-0001] Adams, E. N. , Clark, H. M. , Galano, M. M. , Stein, S. F. , Grogan‐Kaylor, A. , & Graham‐Bermann, S. (2018). Predictors of housing instability in women who have experienced intimate partner violence. Journal of Intimate Partner Violence, 36, 3459–3481. Advance online publication. 10.1177/0886260518777001 29779458

[jcop22637-bib-0002] Akaike, H. (1974). A new look at the statistical model identification. IEEE Transactions on Automatic Control, 19(6), 716–723.

[jcop22637-bib-0003] Asparouhov, T. , & Muthén, B. (2014). Auxiliary variables in mixture modeling: Three‐step approaches using M plus. Structural Equation Modeling: A Multidisciplinary Journal, 21(3), 329–341.

[jcop22637-bib-0004] Baker, C. K. , Billhardt, K. A. , Warren, J. , Rollins, C. , & Glass, N. E. (2010). Domestic violence, housing instability, and homelessness: A review of housing policies and program practices for meeting the needs of survivors. Aggression and Violent Behavior, 15, 430–439. 10.1016/j.avb.2010.07.005

[jcop22637-bib-0006] Barile, J. P. , Pruitt, A. S. , & Parker, J. L. (2018). A latent class analysis of self‐identified reasons for experiencing homelessness: Opportunities for prevention. Journal of Community & Applied Social Psychology, 28(2), 94–107.

[jcop22637-bib-0007] Barile, J. P. , Pruitt, A. S. , & Parker, J. L. (2020). Identifying and understanding gaps in services for adults experiencing homelessness. Journal of Community & Applied Social Psychology, 30(3), 262–277.

[jcop22637-bib-0008] Breiding, M. J. , Chen, J. , Black, M. C. , & National Center for Injury Prevention and Control, Centers for Disease Control and Prevention . (2014). Intimate partner violence in the United States 2010. file:///C:/Users/DMC254/Downloads/cdc_21961_DS1.pdf

[jcop22637-bib-0009] Breiding, M. J. , & Armour, B. S. (2015). The association between disability and intimate partner violence in the United States. Annals of Epidemiology, 25(6), 455–457. 25976023 10.1016/j.annepidem.2015.03.017 PMC4692458

[jcop22637-bib-0010] Brewin, C. R. , Rose, S. , Andrews, B. , Green, J. , Tata, P. , Mcevedy, C. , Turner, S. , & Foa, E. B. (2002). Brief screening instrument for post‐traumatic stress disorder. The British Journal of Psychiatry, 181(2), 158–162. 12151288 10.1017/s0007125000161896

[jcop22637-bib-0011] Bridges, A. J. , Andrews, III, A. R. , & Deen, T. L. (2012). Mental health needs and service utilization by Hispanic immigrants residing in mid‐southern United States. Journal of Transcultural Nursing, 23(4), 359–368. 22802297 10.1177/1043659612451259 PMC4060822

[jcop22637-bib-0012] Brown, T. A. (2015). Methodology in the social sciences (Confirmatory factor analysis for applied research 2nd ed.). The Guilford Press.

[jcop22637-bib-0013] Cattaneo, L. B. , Stylianou, A. M. , Hargrove, S. , Goodman, L. A. , Gebhard, K. T. , & Curby, T. W. (2020). Survivor‐centered practice and survivor empowerment: Evidence from A research–practitioner partnership. Violence Against Women, 27, 1252–1272. 10.1177/1077801220935196 32664811

[jcop22637-bib-0074] Chandan, J. S. , Thomas, T. , Raza, K. , Bradbury‐Jones, C. , Taylor, J. , Bandyopadhyay, S. , & Nirantharakumar, K. (2019). Intimate partner violence and the risk of developing fibromyalgia and chronic fatigue syndrome. Journal of Interpersonal Violence, 0886260519888515. 10.1177/0886260519888515 31805821

[jcop22637-bib-0014] Clough, A. , Draughon, J. E. , Njie‐Carr, V. , Rollins, C. , & Glass, N. (2014). “Having housing made everything else possible:” Affordable, safe and stable housing for women survivors of violence. Qualitative Social Work, 13(5), 671–688. 10.1177/1473325013503003 25328440 PMC4196210

[jcop22637-bib-0015] Culhane, D. P. , Metraux, S. , & Byrne, T. (2011). A prevention‐centered approach to homelessness assistance: A paradigm shift? Housing Policy Debate, 21(2), 295–315. 10.1080/10511482.2010.536246

[jcop22637-bib-0016] Curran, P. J. , McGinley, J. S. , Bauer, D. J. , Hussong, A. M. , Burns, A. , Chassin, L. , Sher, K. , & Zucker, R. (2014). A moderated nonlinear factor model for the development of commensurate measures in integrative data analysis. Multivariate Behavioral Research, 49(3), 214–231. 25960575 10.1080/00273171.2014.889594 PMC4423418

[jcop22637-bib-0017] Curtis, M. A. , Corman, H. , Noonan, K. , & Reichman, N. E. (2014). Maternal depression as a risk factor for family homelessness. American Journal of Public Health, 104(9), 1664–1670. 25033116 10.2105/AJPH.2014.301941 PMC4151913

[jcop22637-bib-0018] Daoud, N. , Matheson, F. I. , Pedersen, C. , Hamilton‐Wright, S. , Minh, A. , Zhang, J. , & O'Campo, P. (2016). Pathways and trajectories linking housing instability and poor health among low‐income women experiencing intimate partner violence (IPV): Toward a conceptual framework. Women & Health, 56, 208–225. 10.1080/03630242.2015.1086465 26358378

[jcop22637-bib-0019] Davies, J. , & Lyon, E. (2013). Domestic violence advocacy: Complex lives/difficult choices (7). Sage.

[jcop22637-bib-0020] Dias, N. G. , Costa, D. , Soares, J. , Hatzidimitriadou, E. , Ioannidi‐Kapolou, E. , Lindert, J. , Sundin, Ö. , Toth, O. , Barros, H. , & Fraga, S. (2019). Social support and the intimate partner violence victimization among adults from six European countries. Family Practice, 36(2), 117–124. 29788243 10.1093/fampra/cmy042

[jcop22637-bib-0021] El‐Bassel, N. , Gilbert, L. , Wu, E. , Go, H. , & Hill, J. (2005). Relationship between drug abuse and intimate partner violence: A longitudinal study among women receiving methadone. American Journal of Public Health, 95(3), 465–470. 10.2105/AJPH.2003.023200 15727978 PMC1449203

[jcop22637-bib-0022] Evans, D. N. , Blount‐Hill, K. L. , & Cubellis, M. A. (2019). Examining housing discrimination across race, gender and felony history. Housing Studies, 34(5), 761–778.

[jcop22637-bib-0023] Ewing, J. A. (1984). Detecting alcoholism: The CAGE questionnaire. Journal of the American Medical Association, 252(14), 1905–1907. 6471323 10.1001/jama.252.14.1905

[jcop22637-bib-0024] Goodman, L. A. , Thomas, K. , Cattaneo, L. B. , Heimel, D. , Woulfe, J. , & Chong, S. K. (2016). Survivor‐defined practice in domestic violence work: Measure development and preliminary evidence of link to empowerment. Journal of Interpersonal Violence, 31(1), 163–185. 25381271 10.1177/0886260514555131

[jcop22637-bib-0025] Goodman, L. A. , & Smyth, K. F. (2011). A call for a social network‐oriented approach to services for survivors of intimate partner violence. Psychology of Violence, 1(2), 79–92. 10.1037/a0022977

[jcop22637-bib-0026] Gubits, D. , Shinn, M. , Bell, S. , Wood, M. , Dastrup, S. , Solari, C. , Brown, S. , Brown, S. , Dunton, L. , Lin, W. , McInnis, D. , Rodriguez, J. , Savidge, G. , & Spellman, B. , (Abt Associates, Inc. 2015). *Family Options Study: Short‐term impacts of housing and service interventions for homeless families*. U.S. Department of Housing and Urban Development Office of Policy Development and Research. https://www.huduser.gov/portal/portal/sites/default/files/pdf/FamilyOptionsStudy_final.pdf

[jcop22637-bib-0073] Guyon‐Harris, K. L. , Ahlfs‐Dunn, S. , & Huth‐Bocks, A. (2017). PTSD symptom trajectories among mothers reporting interpersonal trauma: Protective factors and parenting outcomes. Journal of Family Violence, 32(7), 657–667.

[jcop22637-bib-0027] Hetling, A. , Dunford, A. , & Botein, H. (2020). Community in the permanent supportive housing model: Applications to survivors of intimate partner violence. Housing, Theory and Society, 37(4), 400–416.

[jcop22637-bib-0028] Hegarty, K. , Sheehan, M. , & Schonfeld, C. (1999). A multidimensional definition of partner abuse: Development and preliminary validation of the Composite Abuse Scale. Journal of Family Violence, 14(4), 399–415.

[jcop22637-bib-0029] Hernández‐Martinez, M. , Serrata, M. J. V. , & Huitrón, K. (2018). Finding a Way. Innovative housing solutions of Latin@ survivors of domestic violence and successful practices of culturally specifc community‐based organizations. National Latin@ Network. Report, 2.

[jcop22637-bib-0030] Holden, L. , Lee, C. , Hockey, R. , Ware, R. S. , & Dobson, A. J. (2014). Validation of the MOS Social Support Survey 6‐item (MOS‐SSS‐6) measure with two large population‐based samples of Australian women. Quality of Life Research, 23(10), 2849–2853. 24962651 10.1007/s11136-014-0741-5

[jcop22637-bib-0031] Iratzoqui, A. , & Cohn, E. G. (2020). The reporting and help‐seeking behaviors of domestic violence victims with criminal backgrounds. Sociology Compass, 14, e12771. 10.1111/soc4.12771

[jcop22637-bib-0032] Jahiel, R. , & Babor, T. F. (2007). Toward a typology of homeless families: Conceptual and methodological issues. In D. J. Rog , C. S. Holupka , & L. C. Patton (Eds.), Characteristics and dynamics of homeless families with children: Final report to the office of the assistant secretary for planning and evaluation, Office of Human Services Policy, U.S. Department of Health and Human Services. US Department of Health and Human Services.

[jcop22637-bib-0033] Jason, L. A. , Olson, B. D. , Ferrari, J. R. , Majer, J. M. , Alvarez, J. , & Stout, J. (2007). An examination of main and interactive effects of substance abuse recovery housing on multiple indicators of adjustment. Addiction, 102(7), 1114–1121. 17567399 10.1111/j.1360-0443.2007.01846.x PMC2976482

[jcop22637-bib-0034] Johnstone, M. , Parsell, C. , Jetten, J. , Dingle, G. , & Walter, Z. (2016). Breaking the cycle of homelessness: Housing stability and social support as predictors of long‐term well‐being. Housing Studies, 31, 410–426.

[jcop22637-bib-0072] Kennedy, A. C. , Adams, A. , Bybee, D. , Campbell, R. , Kubiak, S. P. , & Sullivan, C. (2012). A model of sexually and physically victimized women's process of attaining effective formal help over time: The role of social location, context, and intervention. American Journal of Community Psychology, 50(1–2), 217–228. 22290627 10.1007/s10464-012-9494-x

[jcop22637-bib-0035] Kroenke, K. , Spitzer, R. L. , & Williams, J. B. (2001). The PHQ‐9: Validity of a brief depression severity measure. Journal of General Internal Medicine, 16(9), 606–613. 11556941 10.1046/j.1525-1497.2001.016009606.x PMC1495268

[jcop22637-bib-0036] Kubiak, S. , Sullivan, C. M. , Fries, L. , Nnawulezi, N. , & Fedock, G. (2011). Best practice toolkit for working with domestic violence survivors with criminal histories. Michigan Coalition Against Domestic and Sexual Violence.

[jcop22637-bib-0037] Liang, B. , Goodman, L. , Tummala‐Narra, P. , & Weintraub, S. (2005). A theoretical framework for understanding help‐seeking processes among survivors of intimate partner violence. American Journal of Community Psychology, 36(1–2), 71–84. 10.1007/s10464-005-6233-6 16134045

[jcop22637-bib-0038] Lo, Y. , Mendell, N. R. , & Rubin, D. B. (2001). Testing the number of components in a normal mixture. Biometrika, 88, 767–778.

[jcop22637-bib-0039] Loxton, D. , Powers, J. , Fitzgerald, D. , Forder, P. , Anderson, A. , Taft, A. , & Hegarty, K. (2013). The Community Composite Abuse Scale: Reliability and validity of a measure of intimate partner violence in a community survey from the ALSWH. Journal of Women's Health Issues Care, 2(4). 10.4172/2325-9795.1000115

[jcop22637-bib-0040] Macy, R. J. , & Goodbourn, M. (2012). Promoting successful collaborations between domestic violence and substance abuse treatment service sectors: A review of the literature. Trauma, Violence & Abuse, 13(4), 234–251. 10.1177/1524838012455874 22899704

[jcop22637-bib-0041] Mowbray, C. T. , Jordan, L. C. , Ribisl, K. M. , Kewalramani, A. , Luke, D. , Herman, S. , & Bybee, D. (1999). Analysis of postdischarge change in a dual diagnosis population. Health & Social Work, 24(2), 91–101. 10340159 10.1093/hsw/24.2.91

[jcop22637-bib-0042] Muthén, B. , & Muthén, L. K. (2020). Mplus statistical software program (version 8.5). Muthén, & Muthén. Los Angeles, California.

[jcop22637-bib-0043] Nichols, M. P. (2013). Self in the system: Expanding the limits of family therapy. Routledge.

[jcop22637-bib-0044] Nilsson, S. F. , Nordentoft, M. , & Hjorthøj, C. (2019). Individual‐level predictors for becoming homeless and exiting homelessness: A systematic review and meta‐analysis. Journal of Urban Health, 96, 741–750. 31388823 10.1007/s11524-019-00377-x PMC6814700

[jcop22637-bib-0045] Ogbe, E. , Harmon, S. , Van den Bergh, R. , & Degomme, O. (2020). A systematic review of intimate partner violence interventions focused on improving social support and/mental health outcomes of survivors. PLOS One, 15(6), e0235177. 32584910 10.1371/journal.pone.0235177 PMC7316294

[jcop22637-bib-0046] Olivet, J. , Dones, M. , Richard, M. , Wilkey, C. , Yampolskaya, S. , Beit‐Arie, M. , Joseph, L. , & Center for Social Innovation (2018). SPARC Supporting Partnerships for Anti‐Racist Communities: Phase one study findings. https://center4si.com/wp-content/uploads/2016/08/SPARC-Phase-1-Findings-March-2018.pdf

[jcop22637-bib-0047] Pavao, J. , Alvarez, J. , Baumrind, N. , Induni, M. , & Kimerling, R. (2007). Intimate partner violence and housing instability. American Journal of Preventive Medicine, 32(2), 143–146. 17234488 10.1016/j.amepre.2006.10.008

[jcop22637-bib-0048] Phipps, M. , Dalton, L. , Maxwell, H. , & Cleary, M. (2019). Women and homelessness, a complex multidimensional issue: Findings from a scoping review. Journal of Social Distress and the Homeless, 28, 1–13.

[jcop22637-bib-0049] Postmus, J. L. , Severson, M. , Berry, M. , & Yoo, J. A. (2009). Women's experiences of violence and seeking help. Violence Against Women, 15(7), 852–868. 19458091 10.1177/1077801209334445

[jcop22637-bib-0050] Purtle, J. , Gebrekristos, L. T. , Keene, D. , Schlesinger, P. , Niccolai, L. , & Blankenship, K. M. (2020). Quantifying the restrictiveness of local housing authority policies toward people with criminal justice histories: United States, 2009–2018. American Journal of Public Health, 110(S1), S137–S144. 31967881 10.2105/AJPH.2019.305437 PMC6987923

[jcop22637-bib-0051] Rog, D. J. , & Buckner, J. C. (2007 September). 5‐homeless families and children. In Toward Understanding Homelessness: The 2007 National Symposium, 4, 2.

[jcop22637-bib-0052] Schwarz, G. (1978). Estimating the dimension of a model. The Annals of Statistics, 6(2), 461–464.

[jcop22637-bib-0053] Sclove, S. L. (1987). Metric considerations in clustering: implications for algorithms. In *Multivariate Statistical Modeling and Data Analysis* (pp. 163–186). Springer.

[jcop22637-bib-0054] Shaw, R. (2020). Generation priced out: Who gets to live in the New Urban America, with a New Preface. University of California Press.

[jcop22637-bib-0055] Smith, P. H. , Homish, G. G. , Leonard, K. E. , & Cornelius, J. R. (2012). Intimate partner violence and specific substance use disorders: Findings from the National Epidemiologic Survey on Alcohol and Related Conditions. Psychology of Addictive Behaviors, 26(2), 236–245. 10.1037/a0024855 21823768 PMC3883081

[jcop22637-bib-0056] Spencer, C. M. , Stith, S. M. , & Cafferky, B. (2019). Risk markers for physical intimate partner violence victimization: A meta‐analysis. Aggression and Violent Behavior, 44, 8–17.

[jcop22637-bib-0057] Spitzer, R. L. , Kroenke, K. , Williams, J. B. , & Löwe, B. (2006). A brief measure for assessing generalized anxiety disorder: The GAD‐7. Archives of Internal Medicine, 166(10), 1092–1097. 16717171 10.1001/archinte.166.10.1092

[jcop22637-bib-0058] Stylianou, A. M. , & Pich, C. (2019). Beyond domestic violence shelter: Factors associated with housing placements for survivors exiting emergency shelters. Journal of Interpersonal Violence, 886260519858393. 10.1177/0886260519858393 31246141

[jcop22637-bib-0059] Sullivan, C. M. (2011). Evaluating domestic violence support service programs: Waste of time, necessary evil, or opportunity for growth? Journal of Aggression and Violent Behavior, 16, 354–360.

[jcop22637-bib-0060] Sullivan, C. M. , Bomsta, H. , & Hacskaylo, M. (2019). Evidence that flexible funding is a promising strategy to prevent homelessness for survivors of intimate partner violence: A longitudinal pilot study. Journal of Interpersonal Violence, 34(14), 3017–3033. 10.1177/0886260516664318 27520017

[jcop22637-bib-0061] Sullivan, C. M. , López Zerón, G. , Bomsta, H. , & Menard, A. (2019). ‘There's just all these moving parts:' Helping domestic violence survivors obtain housing. Clinical Social Work Journal, 47(2), 198–206. 10.1007/s10615-018-0654-9

[jcop22637-bib-0062] Sullivan, C. M. , & Olsen, L. (2016). Common ground, complementary approaches: Adapting the Housing First model for domestic violence survivors. Housing and Society, 43(3), 182–194. 30637331 10.1080/08882746.2017.1323305 PMC6310448

[jcop22637-bib-0063] Sullivan, C. M. , & Virden, T. (2017). An eight state study on the relationships among domestic violence shelter services and residents' self‐efficacy and hopefulness. Journal of Family Violence, 32, 741–750. 10.1007/s10896-017-9930-7

[jcop22637-bib-0064] Sullivan, C. M. , & Virden, T. (2017). Interrelationships among length of stay in a domestic violence shelter, help received, and outcomes achieved. American Journal of Orthopsychiatry, 87(4), 434–442. 10.1037/ort0000267 28394154

[jcop22637-bib-0065] Sullivan, C. M. (2018). Understanding how domestic violence support services promote survivor well‐being: A conceptual model. Journal of Family Violence, 33, 123–131. 10.1007/s10896-017-9931-6 29367804 PMC5760592

[jcop22637-bib-0066] Theodos, B. , Popkin, S. J. , Parilla, J. , & Getsinger, L. (2012). The challenge of targeting services: A typology of public‐housing residents. Social Service Review, 86(3), 517–544.

[jcop22637-bib-0067] Thomas, K. , Messing, J. T. , Ward‐Lasher, A. , & Bones, A. (2020). No easy decisions: Developing an evidence‐informed process to allocate housing choice vouchers to survivors of intimate partner violence. Housing Policy Debate, 30(5), 783–805.

[jcop22637-bib-0068] U.S. Department of Health and Human Services . (2013). Expanding Opportunities to House Individuals and Families Experiencing Homelessness through the Public Housing (PH) and Housing Choice Voucher (HCV) Programs: Questions and answers. https://www.hud.gov/sites/documents/PIH2013-15HOMELESSQAS.PDF

[jcop22637-bib-0069] Vermunt, J. K. (2010). Latent class modeling with covariates: Two improved three‐step approaches. Political Analysis, 18(4), 450–469.

[jcop22637-bib-0070] Zweig, J. M. , & Burt, M. R. (2007). Predicting women's perceptions of domestic violence and sexual assault agency helpfulness: What matters to program clients? Violence Against Women, 13, 1149–1178. 10.1177/1077801207307799 17951590

